# Insights into the Potential of the Atlantic Cod Gut Microbiome as Biomarker of Oil Contamination in the Marine Environment

**DOI:** 10.3390/microorganisms7070209

**Published:** 2019-07-22

**Authors:** Juline M. Walter, Andrea Bagi, Daniela M. Pampanin

**Affiliations:** 1 Department of Chemistry, Bioscience and Environmental Engineering, Faculty of Science and Technology, University of Stavanger, NO-4036 Stavanger, Norway; 2 NORCE Norwegian Research Centre AS, 5008 Bergen, Norway

**Keywords:** gut, prokaryote, animal, marine, microbiome

## Abstract

Background: Microorganisms are widespread in all environments, including in and on animal bodies. The gut microbiome has an essential influence on fish health, and is affected by several persistent and harmful organic and inorganic contaminants. Considering the shifts in gut microbiota composition observed in those studies, we hypothesized that certain microbial groups in the gut can serve as indicators of pollution. To test this hypothesis, we explored the possibility of identifying key microbial players that indicate environmental contamination. Methods: Published 16S rRNA gene amplicon sequencing data generated from the gut microbiota of Atlantic cod caught in geographically different Norwegian waters were used for bacterial diversity comparison. Results: Different microbiomes were identified between the northern Norway and southern Norway samples. Several bacterial genera previously identified as polycyclic aromatic hydrocarbon degraders were present only in the samples collected in the southern Norway area, suggesting fish contamination with oil-related compounds. Conclusions: The results contribute to the identification of bacterial taxa present in the Atlantic cod gut that indicate fish exposure to contaminants in the marine environment.

## 1. Introduction

Approximately 10^8^ microbial cells populate the fish digestive tract, influencing the development, metabolism, immune system, ecology, and evolution of the host [1,2,3,4]. Gut microbial communities have a pivotal role on how fish respond to environmental stressors, and specific bacteria (holding specific genomic repertoire) might be used as a proxy to evaluate the fish health condition and the environmental contamination status. Most studies have examined microbial community changes in commercially relevant fish species with the aim of better understanding how gut microbiota can be modulated to produce healthier fish in aquaculture settings [5,6,7]. However, in the past years, interest in the effect of pollutants on the gut microbiota and their interplay with the host in responding to these has grown as well. Few studies have addressed such questions employing high-throughput amplicon sequencing, nor have many so far shown shifts in community composition upon the laboratory exposure of various fish species to contaminants, including pentachlorophenol, polybrominated diphenyl ethers, titanium dioxide nanoparticles, bisphenol A, and crude oil [8,9,10,11].

Offshore oil production on the Norwegian continental shelf and the shipping of crude oil pose continuous risks of exposing the marine ecosystem to oil contamination. Norway is the largest producer of oil and gas among all the 16 OSPAR (Oslo-Paris commission for protecting and conserving the North-East Atlantic and its resources) contracting parties [12], and has increased the total oil gross production in the last few years (15% between 2012–2016) [13], with a possibility of expansion after recent new discoveries of hydrocarbon reserves and wells (particularly in the Arctic region) [14]. Short-term and long-term impacts over habitats and populations are likely to increase with the continued production of petroleum-derived compounds. Therefore, it is of particular interest to monitor the presence of polycyclic aromatic hydrocarbons (PAHs), which are common constituents of crude oil complex mixtures originating from natural sources and anthropogenic activities [15,16]. PAHs have been circulating through biogeochemical cycles for millions of years by the occurrence of natural seeps [17]. Nevertheless, anthropogenic activities have considerably increased PAH levels in the ecosystems [18], and several of them feature on the European Union and the United States lists of priority pollutants to monitor in aquatic and terrestrial ecosystems [19,20], due their carcinogenic, mutagenic, and toxic effects in biota [21,22,23,24,25]. In fish, crude oil–PAHs and residual fuel oils exposure promote several organs disorders, such as toxic hepatic lesions [26] and heart failure [27,28]. The cardiotoxicity effect leads to the direct disruption of fish cardiac function, cardiac edema, arrhythmia, and impaired swimming performance [28,29,30,31,32]. Furthermore, controlled experiments have shown that oil exposure-induced immunosuppression increases susceptibility to bacterial infection and high mortality rates [33]. 

Atlantic cod (*Gadus morhua*) is commonly used as a bioindicator species for monitoring the presence and effects of contaminants in the marine environment [34]. It has been selected in our study due to its commercial value and its use in ecotoxicological studies and monitoring surveys [35,36,37,38,39,40]. Different biomarkers have been successfully used to trace PAH compounds in fish organs, greatly improving the knowledge of PAHs effects and persistence. While the mechanisms of PAH detoxification through liver enzyme activities in fish are well established and used as biomarkers of pollution [41], less is known about the fate and effect of PAH metabolites entering the intestinal tract of the fish via the bile content. 

High-throughput sequencing technologies have contributed to increasing our knowledge regarding the identities of microbial key players growing in the presence of petroleum-derived PAH mixtures [42,43,44,45,46,47], as well as identifying which metabolic activities of resident microbes may exacerbate or mitigate the toxic effects of these metabolites. Atlantic cod gut microbiota was shown to be affected by crude oil exposure at 0.05 mg·L^−1^ and 0.1 mg·L^−1^ in a laboratory study [11]. The results of this high-throughput sequencing analysis suggested that a set of bacterial taxa that are present in the gut of Atlantic cod can be used as novel indicators of PAH contamination. The microbiome sequence analysis was the selected approach in our work to trace contamination. The sequence analysis strategy is faster and highly sensitive in comparison with traditional methodologies, bringing more valuable detailed information besides confirming former results obtained using, e.g., the traditional set of fish health biological markers [48,49].

Here, we explore the potential of specific microbial taxa that are present in the Atlantic cod gut microbiome as oil contamination markers using publicly available data. Our goals were: (i) to perform an overall comparison of the microbial community composition of gut samples collected in a presumably pristine northern area of Norway and in a more anthropogenic impacted one located in southern Norway; and (ii) to analyze the relative abundance of known marine hydrocarbon degraders bacteria in the different gut samples.

## 2. Materials and Methods 

### 2.1. Samples

Publicly available sequencing data were obtained from wild cod caught in northern Norway (Lofoten and Sørøya locations) and in southern Norway (Kvitsøy, Rogaland) (Table 1). Cod from the southern Norway area were maintained in laboratory conditions and only fish exposed to no oil and low concentration of oil (0.01 ppm) were used in this study, as these were highly similar according to a preliminary analysis [11], and the exposure levels were the closest to real environmental contamination conditions. A total of seven samples were obtained from Riiser et al. [50] (northern Norway: four Lofoten, and three Sørøya) and six were obtained from Bagi et al. [11] (southern Norway: three concentration samples with no oil, and three with low oil). Both datasets were based on high-throughput Illumina sequencing of the 16S rRNA V4 region, and were generated based on fish intestinal content and mucosa. A limited amount of Atlantic cod gut microbiome samples using the same sequencing technology and the same 16S rRNA region is publicly available at present. The use of 16S rRNA sequence data allows the analysis of the prokaryote components, which are directly influenced by environmental changes [51]. Here, we focused on the microbial PAH-degraders (Bacteria domain) found in the gastrointestinal organ.

### 2.2. Quality Filtering of Sequence Data and Analysis

Raw sequences data were downloaded from the European Nucleotide Archive (PRJEB22384 for [50] and PRJEB21667 for [11]) database and processed under the same methodology to minimize bias. Sequencing reads of 16S rRNA gene were checked for quality with FastQC v. 0.11.2 [52] before being filtered and processed through the DADA2 package v. 1.6 [53], implemented in R software environment v. 3.6.0 [54], to generate amplicon sequence variants (ASVs) corresponding to high-quality sequences at single-nucleotide resolution. ASV table is generated after several steps: quality check, trimming, filtering (Supplementary Figure S1a,b), dereplicating sequences, learning error rates (Figure S2a,b), removing sequences potentially containing errors (denoising), merging paired-end reads as contigs, screening contigs for mismatches to reduce errors, sequence variant abundance identification, and removing chimeric sequences (using the “bimera” method). Each ASV was taxonomically classified to the closest reference sequence of the Silva bacterial database (release 132) [55,56]. The assigned results were classified at domain to genus level.

Alpha diversity was applied to analyze taxa diversity in a sample through the number of observed ASVs, Chao1 richness, Shannon diversity index, Simpson diversity index, inverse Simpson, and ACE. Relative taxa abundances were represented in stacked barplots. To analyze how closely related the samples were to each other, beta diversity analyses were determined based on the Bray–Curtis dissimilarities metric and weighted UniFrac phylogenetic distances metric. Both distances were visualized using the ordination method principal coordinate analysis (PCoA) into two-dimensional plots. Also, Ward’s hierarchical clustering method based on Euclidean distance was applied to interpret the distance matrix using average linkages. Differences in the samples’ beta diversity were measured using the permutational multivariate analysis of variance (PERMANOVA) test with the *adonis* function from the vegan package v. 2.5-5 [57] in R. The Microbiome Analyst [58] tool was used to perform the diversity and compositional analysis, as well as comparative analysis based on the ASVs table from the 16S rRNA sequencing data used in the present study. The ASVs abundance table was filtered, and a normalization step using the minimum library size and data transformation based on the relative log expression (RLE) method was used to account for the compositional differences. To identify differences in taxa abundances of fish gut samples from two different locations, we used differential expression analysis for sequence count data v. 2 (DESeq2) [59].

The ASVs abundance table corresponds to the traditional operational taxonomic units (OTUs) table. Although the ASV-based method has been shown to generate more accurate results for analysis of 16S rRNA [60,61], we also inferred the taxonomical composition of the samples using OTUs. 16S rRNA gene sequences were filtered and processed using the quantitative insights into microbial ecology (QIIME) v. 1.8 [62] that uses the USEARCH algorithm v. 9.2 [63] to find the closest taxa in the reference database. To process raw Illumina data, paired-end reads were merged with a minimum overlap of 10 bp and a 5% error rate in the overlapping bases. Singletons were removed. Joined reads were trimmed by quality, removing low complexity sequences with a Phred score less than 25, as well as length and ambiguous bases. Then, samples were then screened for chimeric sequences using the default parameters in UCHIME algorithm v. 2011-11-02 [64], and the detected chimerae were removed. Merged sequences that passed quality filtering thresholds were subsequently clustered into OTUs, based on 97% identity using the UPARSE command [65] in USEARCH. Taxonomic information was provided using the Silva bacterial database (release v.119), and the assigned results were classified at the domain to genus level.

## 3. Results

The resulting high-quality non-chimeric-reads table retained 392 ASVs, which were used for downstream analysis. Overall, the observed error rates from the learning error rates step tracked well with the estimated error rates (Figure S2a,b), indicating a suitable quality control based on Phred quality scores (Figure S1a,b).

### 3.1. Overview of the bacterial community profiles 

Richness estimates (Chao1, ACE, Fisher, and number of ASVs) and alpha diversity were calculated as Shannon and Simpson’s indices, showed fluctuations across fish gut from northern Norway and southern Norway (Figure S3). In addition, fish gut from northern Norway had a lower microbial community richness compared to the fish gut from southern Norway (Figure S3). The same was observed when analyzing OUT-based results (data not shown).

**Figure 1:**
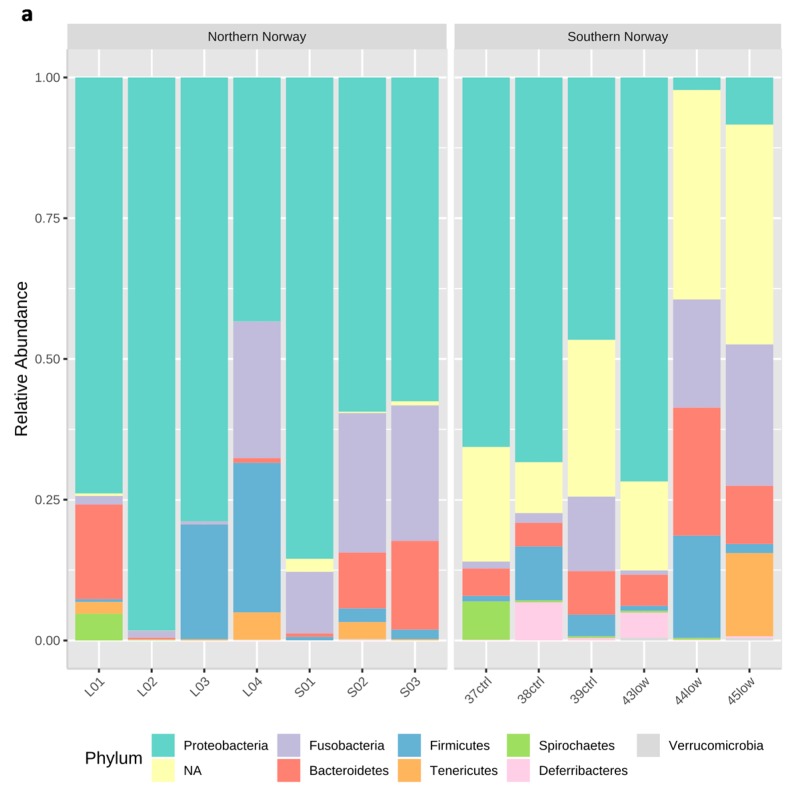

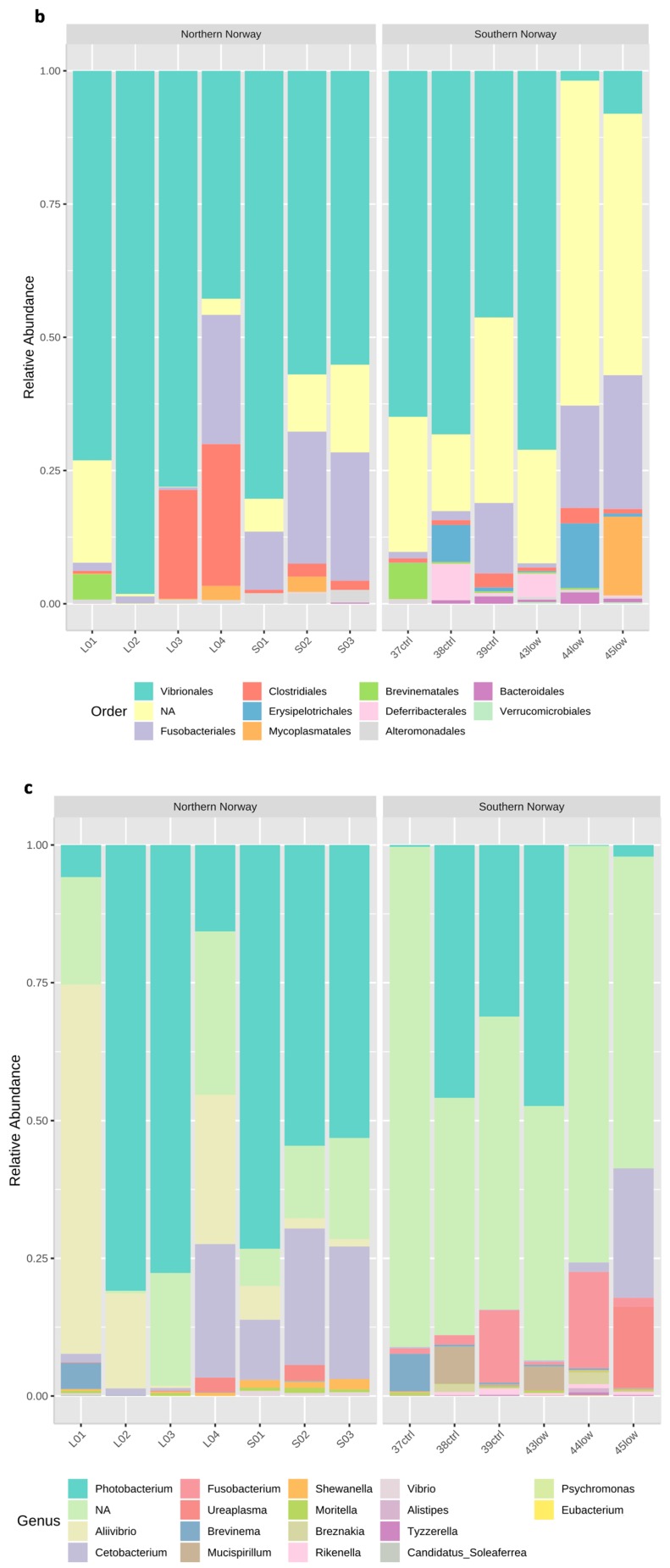
Microbial community composition in Atlantic cod gut samples from geographically different areas. Relative abundance of the sequences is expressed as percentage (%) and are presented at the phylum level (**a**), order level (**b**), and genus level (**c**).

Firmicutes, Proteobacteria, Bacteroidetes, and Fusobacteria were the four most abundant phyla in all the samples (Figure 1a). There were multiple specific differences in the proportions of the relative abundance of various taxa from phylum to genus level when comparing the northern and southern samples (Figure 1a–c and Figure S4). Whereas for the area designed as pristine, i.e., northern Norway, Fusobacteria and Proteobacteria were the most abundant, followed by Firmicutes and Bacteroidetes. In the fish from southern Norway, we observed the dominance of Firmicutes, followed by Proteobacteria, Bacteroidetes, and Fusobacteria. Vibrionales was the most abundant order identified (Figure 1b). The Photobacterium genus was highly frequent in the fish gut from northern Norway (Figure 1c) and notably, non-annotated ASVs could be detected specially in the fish gut from southern Norway (Figure 1c).

**Figure 2:**
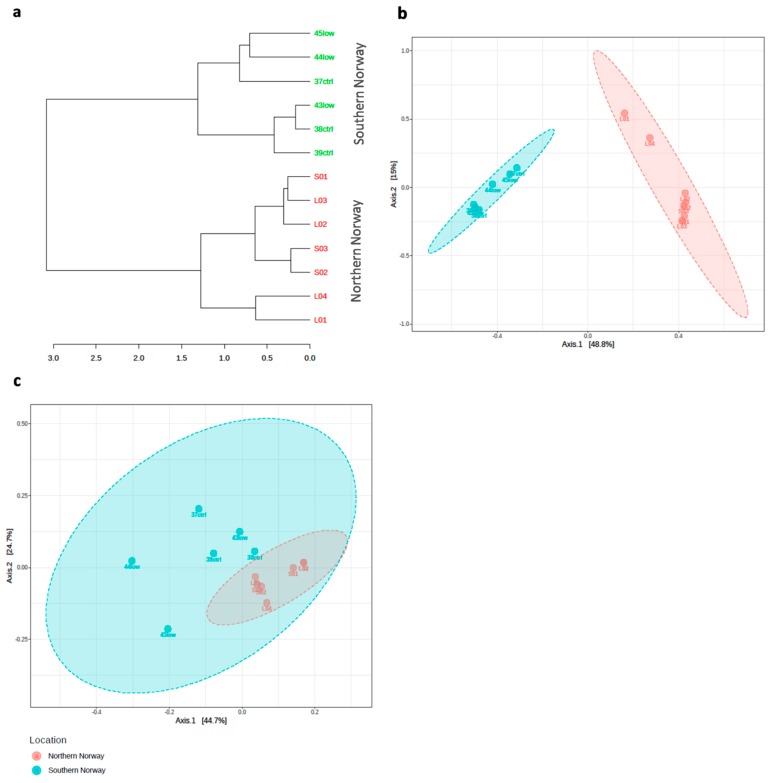
Microbial community composition in Atlantic cod gut samples from northern and southern Norway. (**a**) Hierarchical clustering of amplicon sequence variant (ASVs) abundances generated through Bray–Curtis distance metric and Ward’s linkage method. (**b**) Principal coordinate analysis (PCoA) ordination of all the samples based on the beta diversity analysis of ASV abundances generated using the Bray–Curtis distance metric. (**c**) Nonmetric multidimensional scaling (NMDS) beta diversity ordination of individual samples calculated with weighted UniFrac distances. Oil exposure treatment codes: ctrl = control (no oil), low = low concentration of oil [11]; L = Lofoten; S = Sørøya.

A hierarchical clustering of samples based on a beta diversity analysis of ASV abundances showed cluster relationships between samples, indicating differences in bacterial composition between the northern Norway and southern Norway samples (Figure 2a). This hypothesis is supported by the ordination analysis using PCoA on Bray–Curtis (Figure 2b) and PCoA on ordinated weighted UniFrac distances of individual samples across locations (Figure 2c), where the PERMANOVA results displayed that location accounts for a substantial variation in beta diversity (*R*^2^ = 0.467; *p* < 0.002). The two first axes of the Bray–Curtis distance metric-based PCoA plot explained 63.8% of the taxonomic variation among the microbiomes, while determining by a phylogenetic weighted UniFrac distance metric-based PCoA plot explained 69.4% of the taxonomic variation. The samples from northern Norway clustered together, and samples from southern Norway tended to form a separate group (Figure 2a–c). Location has been observed to influence gut microbial beta diversity. The ordinated beta diversity analysis based on the ASV abundance supports the hypothesis that gut microbiomes from northern Norway fish differ from the gut microbiomes from southern Norway fish (Figure 2a–c).

### 3.2. Differences in Most Abundant Gut Microbiota

**Figure 3:**
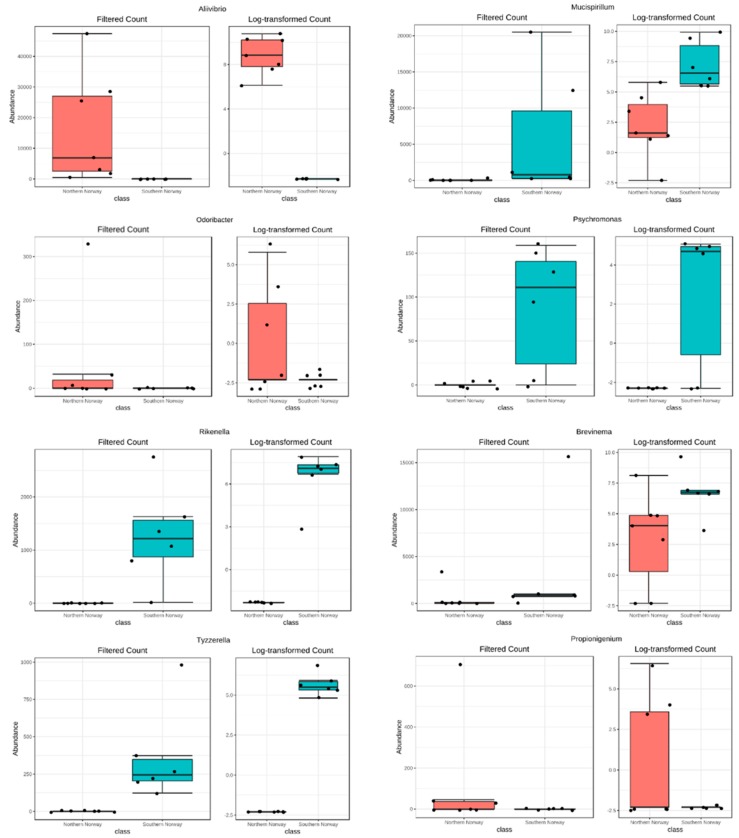

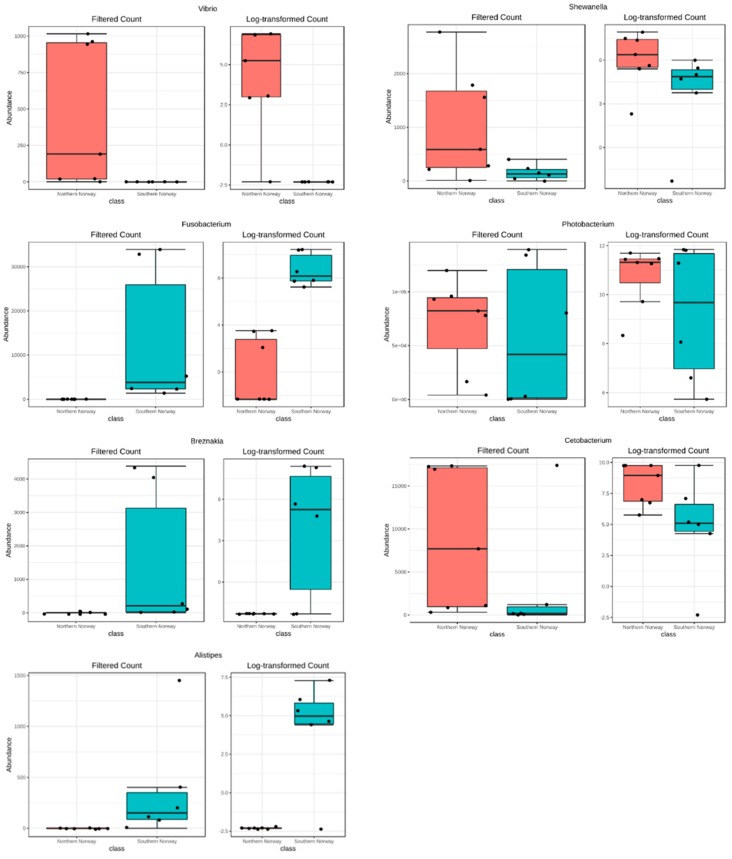
Differential abundance of bacteria in Atlantic cod gut samples from northern and southern Norway. Wald tests of log2 fold differential abundances of amplicon sequence variants (ASVs). Sixteen genera revealed significant features, based on *p* values, with differential abundance.

Wald tests of log2 fold differential abundances of ASVs revealed significant fluctuations in the abundances of several genera (Figure 3). A total of 16 genera with significant features showed differential abundance, and among them, 14 were ranked among the 22 most abundant genera (Figure 1c). Most of the genera that were ranked as significant, based on a *p* value of 0.05, through differential analysis of DESeq2 were located in southern Norway (Figure 3), such as *Rikenella*, *Tyzzerella*, *Fusabacterium*, *Breznakia*, *Alistipes*, *Mucispirilum*, and *Psychromonas*.

### 3.3. Contribution of PAH Degraders Bacteria

A total of 65 bacterial genera, distributed in 24 orders and four phyla (Table S1) that had been previously identified as PAH degraders [12] were found in all the gut microbiota samples. The presence of PAH degraders bacteria was presented here as OTUs results. Previous work [11] reported eight significantly different OTUs in their relative abundance in the gut microbial community of fish exposed to 0.05 mg·L^−1^ and 0.1 mg·L^−1^ of dispersed crude oil. Their presence and abundance were analyzed in the gut microbiomes. Members of Deferribacteraceae responded, increasing their relative abundance with rising oil exposure levels. No Deferribacteraceae sequences were detected in the gut of fish from the northern Norway areas. Analyzing the potential biomarker taxa from [11], *Alistipes* (*p* = 0.04), *Anaerotruncus* (*p* = 0.01), *Parabacteroides* (*p* = 0.03), *Porphyromonas* (*p* = 0.02), and *Ruminococcus* (*p* = 0.01) genera were significantly different in the relative abundance between southern and northern Norway fish gut microbiomes, according to the analysis performed in this study. In addition, *Ornithobacterium* (*p* = 0.05), *Pedobacter* (*p* = 0.02), *Psychromonas* (*p* = 0.02), *Lactobacillus* (*p* = 0.02), and *Salinivibrio* (*p* = 0.02) were also differentially abundant between those geographic areas. Members of known marine hydrocarbon-degrading groups, namely the Oceanospirillales and Thiotrichales orders, were also present through the *Alcanivorax* and *Cycloclasticus* genera [66,67]. Sequences annotated as *Novosphingobium*, *Sphingobium*, and *Sphingomonas*, potentially representing alphaproteobacterial PAH degraders, were also present only in the samples from fish collected in southern Norway [68]. Although these genera were not identified as significant through the ASVs method, with the exception of *Alistipes* (Figure 3), the presence of these bacteria should be considered, suggesting them as an important signal for potential oil-degradation contributors.

## 4. Discussion

Pollutants present in the environment (e.g., persistent organic pollutants such as PAHs) are becoming strong and pivotal factors contributing to shape the individual’s gastrointestinal microbiotype [69]. Therefore, the monitoring of the microbial communities in the gut of marine species as sentinel organisms has the value of indicating the habitat health condition. Bacteria with the potential to degrade hydrocarbons, including PAHs, express genes for oxygenases or peroxidases, as found in *Pseudomonas* (*alk*B, alkane monooxygenase; and *ndo*B, naphthalene monooxygenase), *Rhodococcus* (*alk*B1 and *alk*B2), *Mycobacterium* (*nid*A, pyrene dioxygenase), *Cycloclasticus* (*rhd*, hydroxylating dioxygenases), and *Stenotrophomonas* (*sdr*, dehydrogenase reductase). These genes take part in intricate pathways for the degradation of PAHs and other hydrocarbons, potentially doing so in the fish gut as well.

Although some genera showed significant differences in the relative abundance between the southern and northern Norway samples, results analyzed for false discovery rate by Benjamini–Hochberg tests were all above the corrected *p* value. Nevertheless, the presence and the likely role held by specific bacteria in the fish gut of the two geographically different populations verified in the present study should not be neglected. That is why we were motivated to interpret the results beyond the traditional *p* value. Although we know that geographic location, salinity, seasonality and developmental stage are factors that could influence differences in the gut microbiome of fish, as well as dietary input in the aquaculture conditions [70,71,72,73], the presence of well-known bacteria that degrade PAHs compounds reflects the potential presence of oil in surrounding waters. Bacterial genera such as *Novosphingobium*, *Sphingobium*, and *Sphingomonas* present only in the southern Norway fish gut samples might suggest exposure to oil-related compounds, since these Alphaproteobacteria members hold genes that are involved in the degradation of PAHs [74,75]. In addition, OTUs of representative other hydrocarbon-degrading bacteria were present only in the southern Norway fish gut microbiomes, such as *Oleispira antarctica* (Oceanospirillales) and genera *Marinobacter* (Alteromonadaceae), *Rhodococcus* (Actinomicetales), *Microbacterium* (Actinomicetales), *Alcanivorax* (Oceanospirillales), and *Cycloclasticus* (Thiotrichales). For the last two genera, few reads (1–3) were found in some samples from northern Norway. Alkane-degrading bacteria, such as *Alcanivorax* spp., are biological signals of the alkane distribution. A recent study [76] showed that strains belonging to *Alcanivorax* spp., *Marinobacter* spp., and *Planomicrobium* sp. are able to grow on certain nonhydrocarbons, as well as organic compounds such as peptone, glutamic acid, pyruvic acid, sucrose, mannose and others, raising the question of whether there are obligate hydrocarbonoclastic bacteria or not, as previously thought [77,78,79,80]. Although it is important to mention that these bacteria grow on crude oil, *n*-octadecane and phenanthrene, as sole carbon substrates, and they have an important role in the effective removal of hydrocarbons that have spilled in the environment. Indeed, the alkane-degrading *Alcanivorax* spp. were frequently the first to increase in response to oil spill [43,44,81], whereas *Cycloclasticus* spp. are capable of degrading more complex hydrocarbons (including both aromatic and aliphatic hydrocarbon components), usually increasing their population later [46,77,81,82]. *Cycloclasticus* and *Alcanivorax* members, as other PAH degraders, have mechanisms to overcome the low bioavailability of oil droplets. For example, an increased surface hydrophobicity is generated through a biosurfactant produced by *Alcanivorax borkumensis* that facilitates direct contact between bacteria and the contaminant, such as the very hydrophobic *n*-alkane substrate [82]. Such features allow *n*-alkane oxygenase systems, which are located in the membrane, to act directly with their substrates [83]. Species belonging to these genera might act together to degrade PAHs, growing and operating in succession after the fish ingestion of oil droplets through changes in the hydrocarbon supply [84,85,86,87,88].

Acetoclastic iron-reducers that are members of Deferribacteraceae showed significantly different relative abundance in the gut microbiomes of fish exposed to 0.05 mg·L^−1^ and 0.1 mg·L^−1^ of dispersed crude oil compared to samples from fish exposed to 0.01 mg·L^−1^ and no oil [11], and they were absent in all the gut microbiomes from fish collected in northern Norway. The absence of Deferribacteraceae member sequences in the pristine northern Norway microbiomes reinforce the role of these taxa [79,80], and are potential biomarkers of oil pollution. Overall, these genera may be considered key players to guide analyses of fish contamination. Although no statistical significance was found for several taxa, the presence of specific genera only in the southern Norway fish gut microbiomes might suggest a specific role linked to the specific anthropogenic contamination. Shipping, coastal industry activities, and even small-scale oil leakage on the bustling Stavanger fjord (southern Norway) might have an accentuated effect, especially considering the nature of the fjord stagnant waters. Exclusive OTUs could reveal interesting functional effects, including negative displacements, in the host. Considering the contemporary effort on the establishment of an appropriate method to analyze microbiome-sized data, accommodating unique characteristics is under discussion. These include sparsity, sequencing depth variation, and the nonconformity of read counts to theoretical distributions [89]—besides normalization effects—as well as the use and interpretation of traditional *p*-value thresholds [89,90,91].

Shotgun metagenomics and metatranscriptomics surveys are seen as the next step. This approach will help us assess a broader taxon identification, including Fungi and Archaea members potentially involved in PAHs degradation. Furthermore, the functional component might be revealed, allowing the detection of specific metabolic genes involved in different functional preferences for the degradation pathways of PAHs.

## 5. Conclusions

The gut microbiome represents a key determinant to a variety of host metabolic and immune functions, including the reaction to oil pollutant exposure. In this study, we have compared the relative abundance of bacterial communities in the gut of Atlantic cod populations from two geographical areas of the Norwegian Continental Shelf. In addition, we verified the presence of PAH-degrading bacteria in the gut microbiome of fish collected in southern Norway. The presence of hydrocarbonoclastic taxa in the southern Norway fish gut microbiome even in the control samples can be explained by the higher anthropogenic impact in this area, in comparison to the northern Norway area. It reinforces that internal microbiota signatures are associated with PAH contaminants, and certain bacterial taxa can be used as indicators to monitor environmental and fish health. Although the 16S rRNA-based approach provides the composition of microbiomes to the genus level, an increasing number of studies are focusing on functional aspects of the whole community using metagenomics and metatranscriptomics sequencing. Through such approaches, dominant metabolic processes can be explored, and the contribution of the whole prokaryotic and eukaryotic communities can be accessed. Using metagenomics and metatranscriptomics approaches to Atlantic cod gut studies, in the context of monitoring polluted environments, will be the subject of further studies.

## Figures and Tables

**Table 1:** Summary of the main features of the study areas.

Area		Latitude	Longitude	Samples
Northern Norway	Lofoten	68°10′19″ N	13°45′00″ E	4
	Sørøya	70°29′33″ N	22°09′51″ E	3
Southern Norway	Kvitsøy	59°03′52″ N	05°24′19″ E	6

## Supplementary Materials

The following are available online at https://www.mdpi.com/2076-2607/7/7/209/s1. Table S1: Bacterial genera able to grow using hydrocarbons as a sole source of carbon and energy.
