# Supplementary material for: Insights into the Potential of the Atlantic Cod Gut Microbiome as Biomarker of Oil Contamination in the Marine Environment
Source: Microorganisms. 2019 Jul 22;7(7):209. doi: 10.3390/microorganisms7070209 (PMC6680985; doi:10.3390/microorganisms7070209)
Supplement: Supplementary file 1 [file microorganisms-07-00209-s001.zip › Suppl Material.docx]

SUPPLEMENTARY MATERIAL

Insights into the potential of the Atlantic cod gut microbiome as biomarker of oil contamination in the marine environment

Juline M. Walter, Andrea Bagi and Daniela M. Pampanin

**Supplementary Figure S1**

**Supplementary Figure S2**

**Supplementary Figure S3**

**Supplementary Figure S4**

**A**

**B**

**Supplementary Figure S1.** Quality profiles based on Phred quality scores for forward (**a**) and reverse (**b**) sequencing reads. The bases are along the x-axis, and the quality score on the y-axis. The grey-scale heatmap displays the quality score distribution at each read position, while the black underlying heatmap shows the frequency of each score at each base position. The green line is the median quality score at the base position, and the orange lines show the quartiles. Read counts are shown.

**A**

**B**

**Supplementary Figure S2.** Error rates for forward (**a**) and reverse (**b**) reads, inferred through the learning method. Black dots: observed error rates; black lines: estimated error rates; red lines: expected error rates.

**Supplementary Figure S3.** Alpha diversity comparison across Northern Norway and Southern Norway samples. Results for diversity indices of species richness (number of OTUs, ACE, Fisher and Chao estimators) and species evenness (Shannon’s diversity index, Simpson’s evenness) are showed.


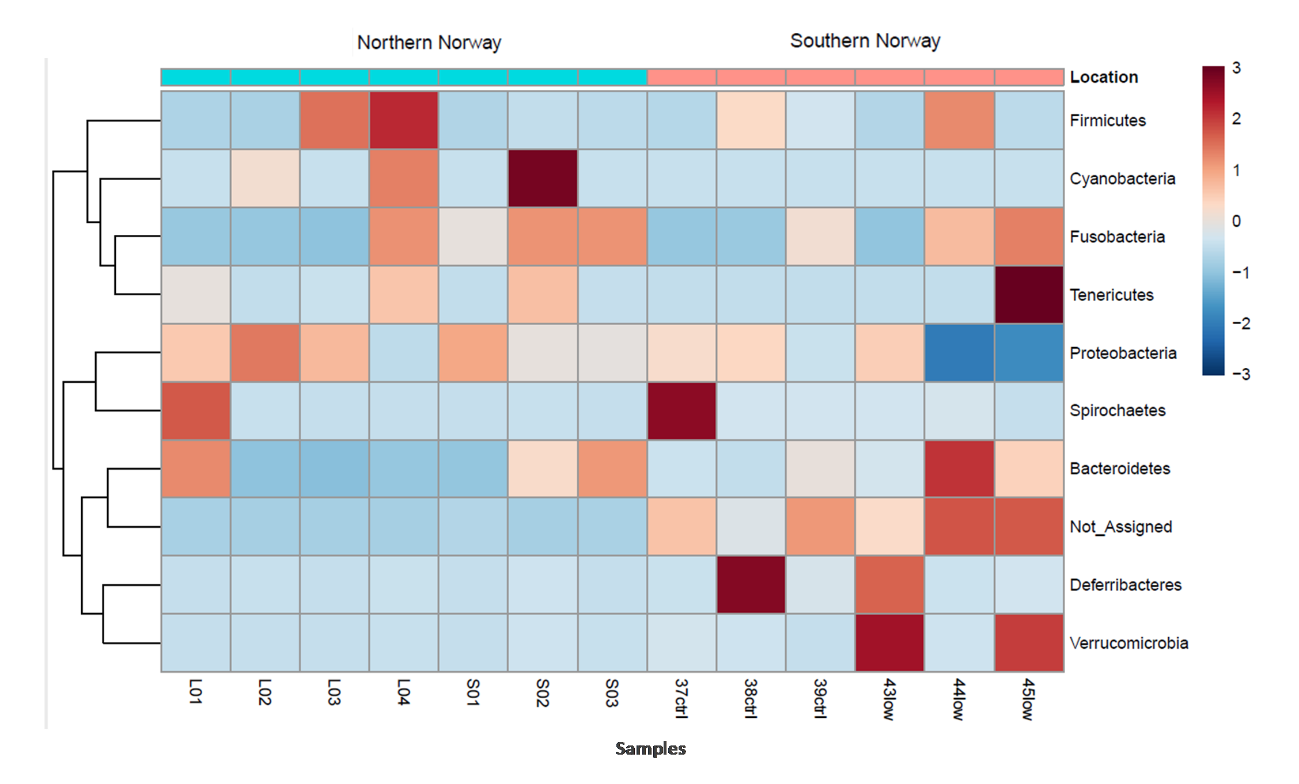


**Supplementary Figure S4.** Similarity of bacterial communities at phylum level associated with fish gut from Northern Norway and Southern Norway. Heatmap hierarchical clustering of amplicon sequence variants (ASVs) using Ward's method showing cluster relationship between similar samples based on Bray-Curtis distance metric. Dendrogram represents similarity of the samples, where each square in the heatmap dendrogram represents the abundance level of a single category in a single sample. Low values might tend towards blue tones while higher values tend to red tones.
